# Genetic enhancement of oil content in potato tuber (*Solanum tuberosum* L.) through an integrated metabolic engineering strategy

**DOI:** 10.1111/pbi.12590

**Published:** 2016-07-11

**Authors:** Qing Liu, Qigao Guo, Sehrish Akbar, Yao Zhi, Anna El Tahchy, Madeline Mitchell, Zhongyi Li, Pushkar Shrestha, Thomas Vanhercke, Jean‐Philippe Ral, Guolu Liang, Ming‐Bo Wang, Rosemary White, Philip Larkin, Surinder Singh, James Petrie

**Affiliations:** ^1^ Commonwealth Scientific and Industrial Research Organisation Agriculture Black Mountain ACT Australia; ^2^ College of Horticulture & Landscape Architecture Southwest University Chongqing China; ^3^ National University of Science and Technology (NUST) Islamabad Islamabad Pakistan; ^4^ State Key Laboratory of Agricultural Microbiology Huazhong Agricultural University Wuhan China

**Keywords:** triacylglycerol, potato, *Solanum tuberosum*, *WRI1*, *DGAT1*, oleosin

## Abstract

Potato tuber is a high yielding food crop known for its high levels of starch accumulation but only negligible levels of triacylglycerol (TAG). In this study, we evaluated the potential for lipid production in potato tubers by simultaneously introducing three transgenes, including *WRINKLED 1* (*WRI1*), *DIACYLGLYCEROL ACYLTRANSFERASE 1* (*DGAT1*) and *OLEOSIN* under the transcriptional control of tuber‐specific (patatin) and constitutive (CaMV‐35S) promoters. This coordinated metabolic engineering approach resulted in over a 100‐fold increase in TAG accumulation to levels up to 3.3% of tuber dry weight (DW). Phospholipids and galactolipids were also found to be significantly increased in the potato tuber. The increase of lipids in these transgenic tubers was accompanied by a significant reduction in starch content and an increase in soluble sugars. Microscopic examination revealed that starch granules in the transgenic tubers had more irregular shapes and surface indentations when compared with the relatively smooth surfaces of wild‐type starch granules. Ultrastructural examination of lipid droplets showed their close proximity to endoplasmic reticulum and mitochondria, which may indicate a dynamic interaction with these organelles during the processes of lipid biosynthesis and turnover. Increases in lipid levels were also observed in the transgenic potato leaves, likely due to the constitutive expression of *DGAT1* and incomplete tuber specificity of the patatin promoter. This study represents an important proof‐of‐concept demonstration of oil increase in tubers and provides a model system to further study carbon reallocation during development of nonphotosynthetic underground storage organs.

## Introduction

The increasing food demand as the result of the growing world population will require substantial growth in future global vegetable oil supply. This has been exacerbated in recent years by the increasing recognition of vegetable oil as a renewable and potentially environmentally friendly alternative energy resource. Current global production of vegetable oil is dominated by just a few crop species including oil palm, soya bean, rapeseed and sunflower, and there is a clear need to create new oil production platforms to help meet growing demand.

Potato (*Solanum tuberosum* L.) is an important stolon tuber crop that has been regarded as the fourth most important staple food crop in the world. The world production of potatoes in 2013 was about 368 million tonnes (FAOSTAT, www.faostat.fao.org). In contrast to oilseed species such as soya bean and rapeseed, potato tuber is rich in starch but very low in oil. Several studies have reported that it is possible to increase oil levels in seeds or leaf tissues by redirecting carbon allocation towards fatty acid biosynthesis and triacylglycerol (TAG) accumulation. This has been achieved by overexpressing the transcription factors and enzymes involved in lipid biosynthesis (Focks and Benning, 1998; Santos Mendoza *et al*., 2005; Vanhercke *et al*., 2014; Vigeolas *et al*., 2007; Zale *et al*., 2016).

In plants, the fatty acid biosynthesis and TAG assembly processes are highly regulated, involving spatial separation of biosynthetic steps between different organelle compartments and the exquisite control of several biosynthetic steps by one or multiple biochemical mechanisms (Harwood, 2005; Weselake *et al*., 2009). *De novo* fatty acid biosynthesis occurs mainly in the plastid where the biotin‐containing enzyme acetyl‐CoA carboxylase (ACCase) catalyses the first committed step by activating acetyl‐CoA to malonyl‐CoA. The malonyl group is then transferred from CoA to an acyl carrier protein (ACP) that serves as the carrier for the growing acyl chain, a step catalysed by a large dissociable multi‐enzyme complex known as fatty acid synthase (FAS). The fatty acids thus synthesised are then exported to the endoplasmic reticulum (ER) where they become precursors for the production of both storage and membrane lipids. In the classical Kennedy pathway, *sn*‐glycerol‐3‐phosphate acyltransferase (GPAT), lysophosphatidic acid acyltransferase (LPAAT) and diacylglycerol acyltransferase (DGAT) catalyse the sequential acylation reactions on a glycerol‐3‐phosphate backbone chain to produce TAG (Weselake *et al*., 2009; Zou *et al*., 1999). TAG synthesised within the ER membrane is then budded off as oil bodies or lipid droplets (LD) that have a single‐layer phospholipid (PL) membrane with the polar head groups in contact with the cytosol and the nonpolar tails in contact with the internal neutral lipids (van Rooijen and Moloney, 1995; Yatsu and Jacks, 1972). Oleosins are small proteins that coat these LDs, helping to stabilise and protect TAG from cytosolic components and withstand the strains of both dehydration and rehydration in seeds (Siloto *et al*., 2006). The amount of oleosin proteins may also play a crucial role in determining the size of LDs (Siloto *et al*., 2006).

Many of the key enzymes involved in fatty acid biosynthesis and lipid assembly have now been isolated, and much effort has been spent in attempts to increase oil content *via* genetic modification. Overexpression of individual enzymes such as *ACCase* in *Nicotiana tabacum* leaves and potato tubers (Klaus *et al*., 2004; Madoka *et al*., 2002), or *DGAT1* in *N. tabacum* leaves (Andrianov *et al*., 2010; Bouvier‐Nave *et al*., 2000) resulted in only limited increases in TAG accumulation. Similarly, reduction in lipid turnover by reducing expression of TAG lipases and enzymes involved in β‐oxidation resulted in only modest TAG accumulation increases (Fan *et al*., 2014; Slocombe *et al*., 2009). Alternative approaches have exploited transcription factors. WRINKLED1 (WRI1) is a transcription factor that was first identified in an Arabidopsis (*Arabidopsis thaliana* L.) mutant producing wrinkled seeds with lower oil content and higher content of sugars than wild type (WT), suggesting that WRI1 is involved in the regulation of carbon flux during seed development (Focks and Benning, 1998). Overexpression of *WRI1* in *wri1* Arabidopsis mutant or *Brassica napus* not only recovered the normal seed appearance but also increased oil content relative to WT Arabidopsis seeds (Cernac and Benning, 2004; Liu *et al*., 2010). Overexpression of *WRI1* in maize (*Zea mays* L.) resulted in 30% higher seed oil content at the expense of starch accumulation (Shen *et al*., 2010). During the preparation process of this manuscript, it was reported that potato tuber overexpressing Arabidopsis *WRI1* alone was able to raise TAG level up to 1% of tuber dry weight (DW) (Hofvander *et al*., 2016).

Recent work has also suggested that the coordinated regulation of the genes involved in transcriptional control of carbon metabolism and those involved in fatty acid and lipid biosynthesis is required for a maximum increase in TAG accumulation in transgenic plants. We have recently reported that the simultaneous overexpression of *WRI1*,* DGAT1* and *OLEOSIN* genes was able to increase TAG content in tobacco leaf to 15% of its DW without severely impacting on plant development (Vanhercke *et al*., 2014). Each of these three genes plays a critical role in directing more carbon flux to fatty acid biosynthesis, TAG assembly and LD biogenesis or protection, respectively. The levels of TAG accumulation achieved by this approach have far exceeded the levels previously reported for engineering TAG yields in plant vegetative tissues, including the recent report in potato tuber expressing *WRI1* alone (Hofvander *et al*., 2016). Such a finding has opened up new possibilities of using high biomass plants as alternative platforms for the production of high energy storage lipids.

The aim of this study was to extend our previous efforts in tobacco leaf to another high biomass plant tissue, with a view to apply our model to one of most extreme carbohydrate‐based storage organ, namely potato tuber. We have examined the potential for lipid production and accumulation in potato tubers by introducing three genes: *WRI1* and *OLEOSIN* under the transcriptional control of a tuber‐specific promoter derived from the potato *PATATIN* gene, and *DGAT1* driven by the constitutive CaMV‐35S promoter. The synergistic functioning of these genes (Vanhercke *et al*., 2013) has resulted in significant increases in TAG and, to a lesser extent, PL and galactolipids (GL).

## Results

### Generation of transgenic potato plants containing transgenes for over‐expression of *WRI1*,* DGAT1* and *OLEOSIN* genes

**Figure 1:**
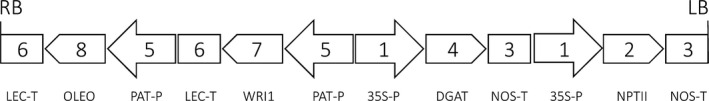
Schematic representation of the construct pJP3506 including the insertion region between the left and right borders. 1, cauliflower mosaic virus 35S promoter with duplicated enhancer region (35S‐P); 2, neomycin phosphotransferase II (NPTII); 3, nopaline synthase terminator (NOS‐T); 4, *Arabidopsis thaliana DGAT1* (DGAT); 5, potato patatin B33 promoter (PAT‐P); 6, *Glycine max* lectin terminator (LEC‐T); 7, *A. thaliana WRI1* (WRI1); 8, *Sesame indicum OLEOSIN* (OLEO).

The binary plasmid construct pJP3506 contained one gene cassette expressing Arabidopsis *DGAT1* (*atDGAT1*) driven by the CaMV‐35S promoter and two gene cassettes expressing Arabidopsis *WRI1* (*atWRI1*) and sesame (*Sesamum indicum* L.) *OLEOSIN* (*siOLEOSIN*), respectively, under the transcriptional control of the tuber‐specific patatin class I promoter B33 (Figure 1). The strong constitutive viral promoter CaMV‐35S was selected for *DGAT1* expression as this promoter had been successfully used to express the *DGAT1* gene in tobacco, resulting in a modest TAG increase without a significant negative impact on plant growth (Bouvier‐Nave *et al*., 2000; Vanhercke *et al*., 2013, 2014). The potato patatin promoter B33 was used as it is highly active in tuber but with low activity in other tissues (Rocha‐Sosa *et al*., 1989). This promoter was selected in order to restrict the alteration of lipid biosynthesis to the tuber only, thereby minimising any potential negative impact on plant development.

Following the transformation of pJP3506 vector in potato (*Solanum tuberosum* cv Atlantic), 178 independent primary transgenic potato lines were selected on kanamycin‐containing media and allowed to grow to maturity in potted soil under glasshouse condition.

### Lipid analysis and molecular assessment of transgene expression in transgenic potato tubers

Assessment of lipid content was carried out in mature tubers harvested from both transgenic and wild‐type (WT) plants that were grown alongside the transgenic plants. Total lipids were extracted and fractionated by thin‐layer chromatography (TLC) for the analysis of TAG, PL and GL by gas chromatography (GC). In the primary screening of transgenic lines, 128 lines were found to contain TAG levels ranging between 0.05 and 4.70% of tuber DW, significantly increased in comparison with the 0.03% in WT. Eight lines with relatively high levels of TAG accumulation were selected to sprout and grow to maturity for further analysis. It is important to note that this was a vegetative reproduction of hemizygous transgenic lines and did not result in the transgenes being genetically fixed through homozygosity.

**Figure 2:**
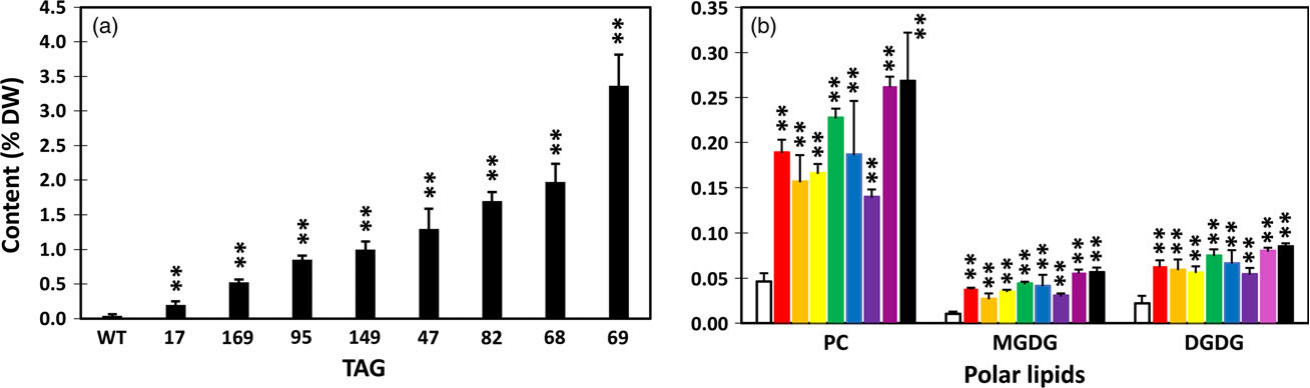
Triacylglycerol (TAG) (a), phosphotidylcholine (PC) and monogalactosyldiacylglycerol (MGDG), digalactosyldiacylglycerol (DGDG) (b) contents in the tubers of wild type (WT) (white bar) and eight selected transgenic potato lines, including #17 (red bar), #169 (orange bar), #95 (yellow bar), #149 (green bar), #47 (blue bar), #82 (indigo bar), #68 (violet bar) and #69 (black bar). Asterisks indicate statistically significant differences between transgenic line and WT using Student's *t*‐test, with a significance threshold of 0.05 (*) and 0.01 (**). Error bars indicate standard deviations.

Among the eight potato transgenic lines, line #69 showed the highest TAG accumulation in tuber with an average level of 3.3% on a DW basis. This is an approximate 100‐fold increase compared with WT (Figure 2a). The same line also accumulated the highest level of phosphotidylcholine (PC) at 0.27% which is 5.8‐fold increase compared with WT; the highest monogalactosyldiacylglycerol (MGDG) at 0.06% which is 5.6‐fold increase relative to WT; and the highest digalactosyldiacylglycerol (DGDG) at 0.86% which is 3.9‐fold increase compared to WT (Figure 2b). The other selected seven lines also showed various levels of increases in TAG, PC, MGDG and DGDG as shown in Figure 2.

**Figure 3:**
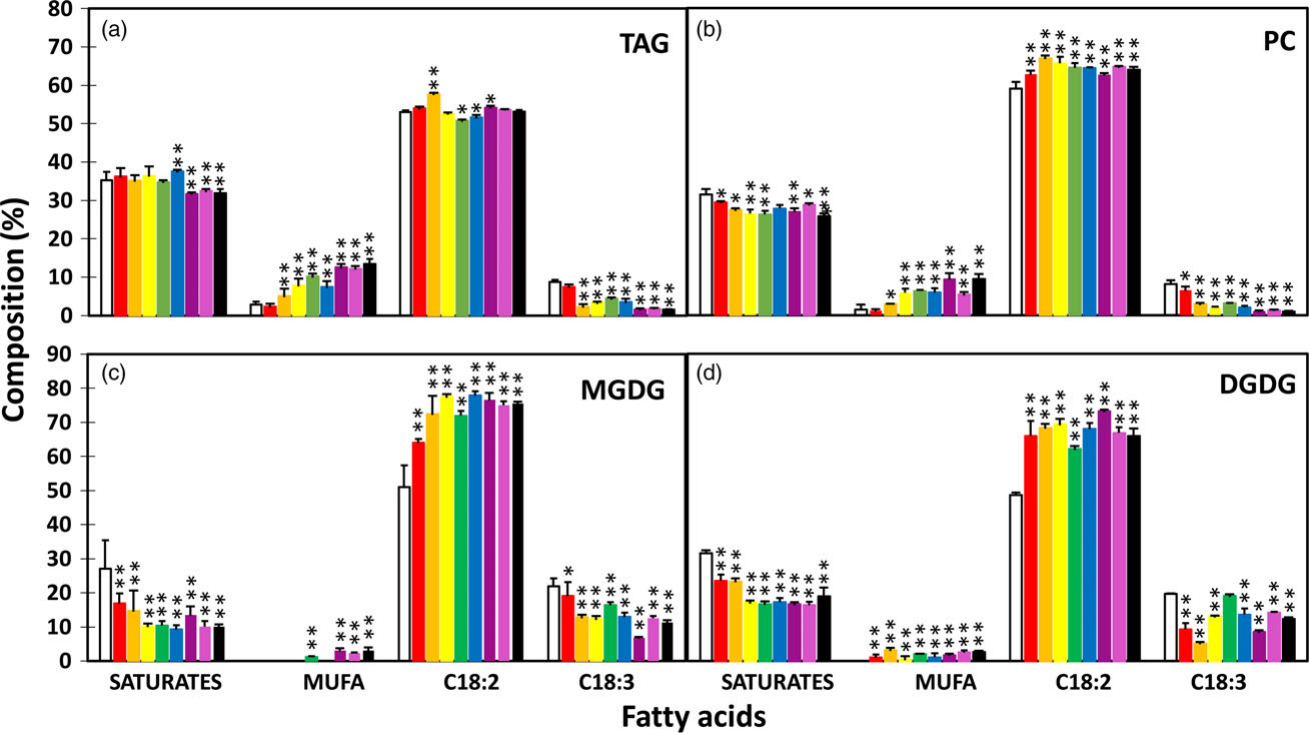
Fatty acid composition in triacylglycerol (TAG) (a), phosphotidylcholine (PC) (b) and monogalactosyldiacylglycerol (MGDG) (c) and digalactosyldiacylglycerol (DGDG) (d) in the tubers of wild type (WT) (white bar) and eight selected transgenic potato lines, including #17 (red bar), #169 (orange bar), #95 (yellow bar), #149 (green bar), #47 (blue bar), #82 (indigo bar), #68 (violet bar) and #69 (black bar). Asterisks indicate statistically significant differences between transgenic line and WT using Student's *t*‐test, with a significance threshold of 0.05 (*) and 0.01 (**). Error bars indicate standard deviations.

The enhanced lipid accumulation was accompanied by altered fatty acid composition in transgenic tubers. The TAG fraction showed significant reductions in the levels of saturated fatty acids and α‐linolenic acid (ALA, C18:3^▵9,12,15^) and significantly increased levels of monounsaturated fatty acids, mostly oleic acid (C18:1^▵9^) and palmitoleic acid (C16:1^▵9^), compared to WT tubers (Figure 3a). A similar trend was also observed in PC, MGDG and DGDG, with the increase in linolenic acid (LA, C18:2^▵9,12^) being more evident than that in TAG (Figure 3b–d).

**Figure 4:**
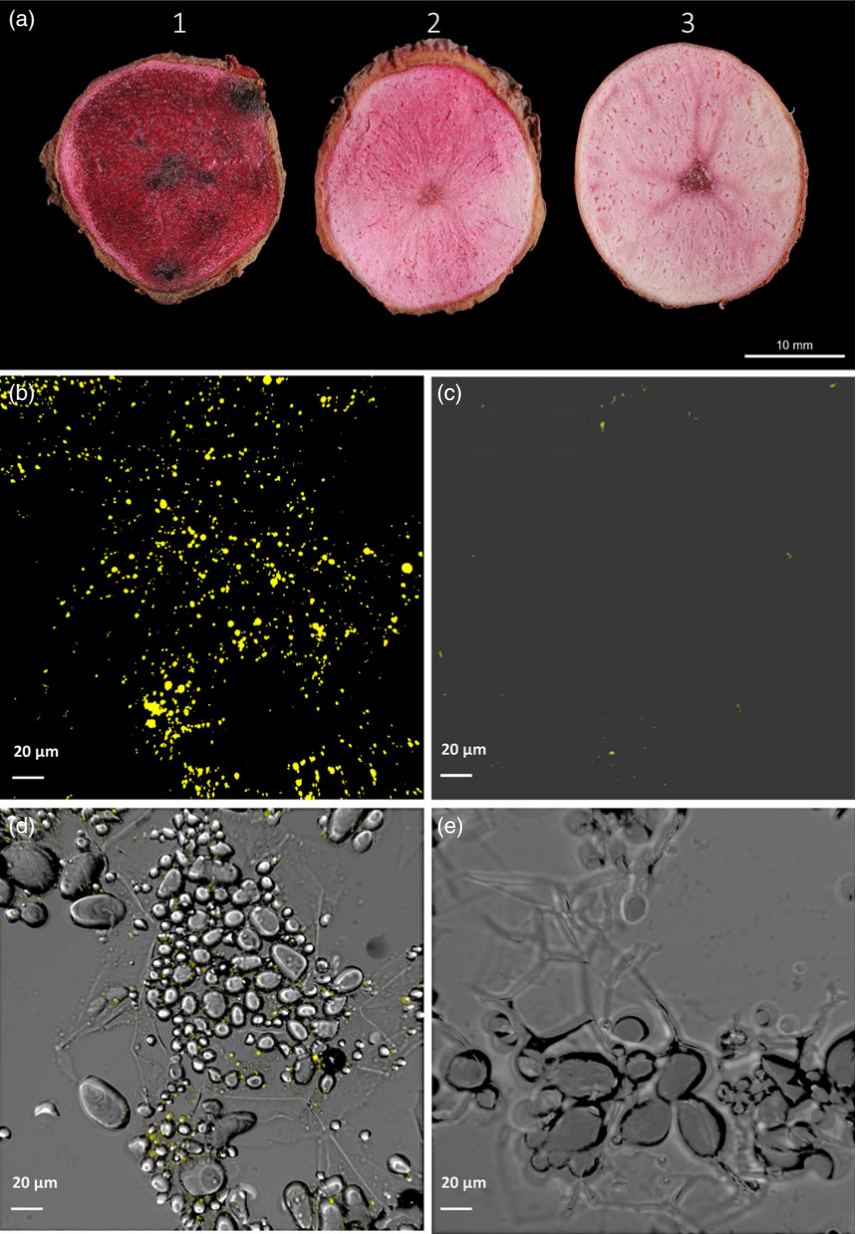
Visualisation of lipid accumulation in potato tubers. (a) Cross section of mature potato tubers showing lipid accumulation as stained with Sudan Red 7B. 1. Line #68; Transgenic potato line #68 showing high triacylglycerol (TAG) accumulation; 2. Line #95 showing moderate level of TAG accumulation; 3. Wild type (WT) showing low level TAG accumulation. (b) Confocal microscopy image of abundant lipid droplets (LDs) following staining with Nile Red in line #68. (c) Confocal microscopy image showing rare appearance of LDs following staining with Nile Red in WT. (d) Bright‐field image of b. (e) Bright‐field image of c.

Cross sections of potato tubers stained with Sudan Red 7B showed enhanced accumulation of neutral lipids in the transgenic tubers in sharp contrast to WT (Figure 4a). It is visibly clear that that the accumulation of neutral lipids in transgenic line #68 is the highest among the three lines examined as indicated by its deepest red stain in tuber. The line #95 tuber showing a moderate increase in neutral lipids was stained in red colour ranging between line #68 and WT control line.

TAG accumulation in transgenic potato tuber (line #68) was visually evident as abundant LDs in tuber cells under confocal microscope following staining with Nile Red (Figure 4b), in clear contrast to WT tubers (Figure 4c). The bright‐field image illustrated that LDs were mostly in close proximity to starch granules in tuber cells (Figure 4d,e).

**Figure 5:**
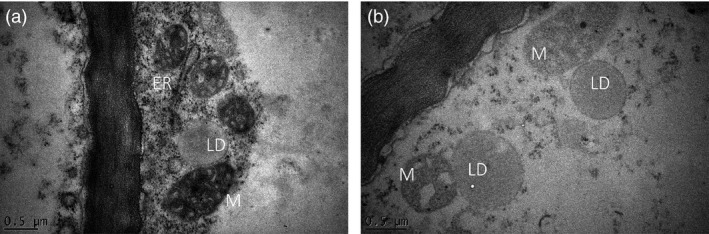
Representative TEM images of transgenic potato line #68, showing lipid droplets (LDs) and their close proximity to endoplasmic reticulum (ER) and mitochondria (M).

Potato tubers of line #68 and WT were also compared by transmission electron microscopy (TEM). The LDs showed typically spherical and ovoid structures, in which the core of neutral lipids, believed to be mostly TAG, was surrounded by presumably a monolayer of PL showing uniform electron density (Figure 5). The sizes of LDs were ranged between 0.5 and 1.5 μm in diameter, which is consistent with the observation of the Nile Red‐stained LDs under confocal microscope. LDs were often found in close proximity with the ER and mitochondria (Figure 5).

**Figure 6:**
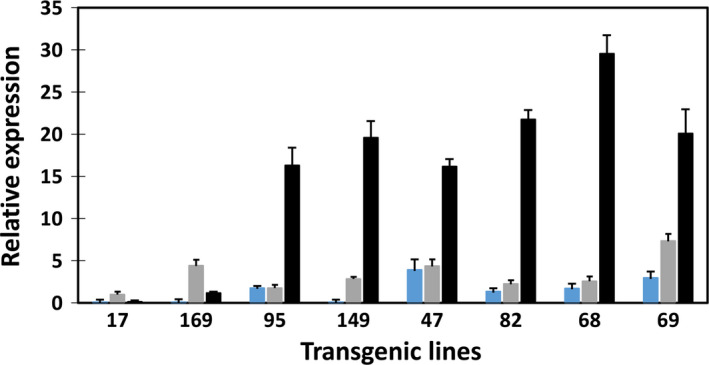
Real‐time qRT‐PCR analysis of transgene expression in developing transgenic potato tubers. *atWRI* (blue bar), *atDGAT1* (shade bar) and *siOLEOSIN* (black bar).

Transgene expression was not detected in WT tubers. The expression levels of the three transgenes in the developing tubers of the eight transgenic lines were assessed by real‐time reverse transcription polymerase chain reaction (qRT‐PCR) (Figure 6). In the transgenic lines, *WRI1* expression was generally lower than that of *DGAT1* and *OLEOSIN*, with the latter showing the highest expression of the three transgenes in all lines except lines #17 and #169. This may indicate that a moderate level of *WRI1* expression is sufficient for the enhancement of TAG accumulation.

### Lipid analysis and molecular assessment of transgene expression in transgenic potato leaves

**Figure 7:**
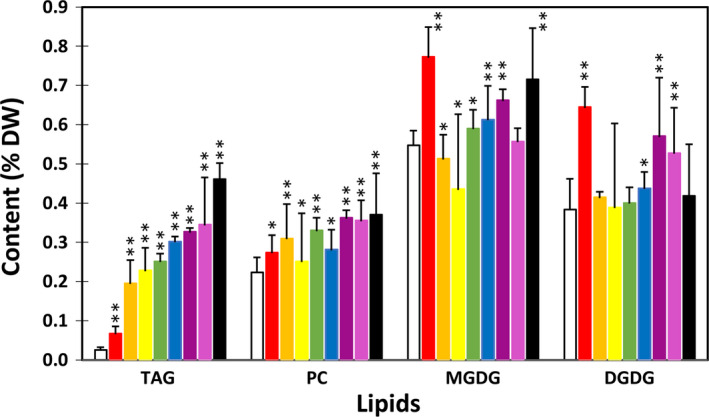
Triacylglycerol (TAG), phosphotidylcholine (PC) and monogalactosyldiacylglycerol (MGDG), digalactosyldiacylglycerol (DGDG) contents in the leaves of wild type (WT) (white bar) and eight selected transgenic potato lines, including #17 (red bar), #47 (orange bar), #82 (yellow bar), #95 (green bar), #169 (blue bar), #69 (indigo bar), #149 (violet bar) and #68 (black bar). Asterisks indicate statistically significant differences between transgenic line and WT using Student's *t*‐test, with a significance threshold of 0.05 (*) and 0.01 (**). Error bars indicate standard deviations.

**Figure 8:**
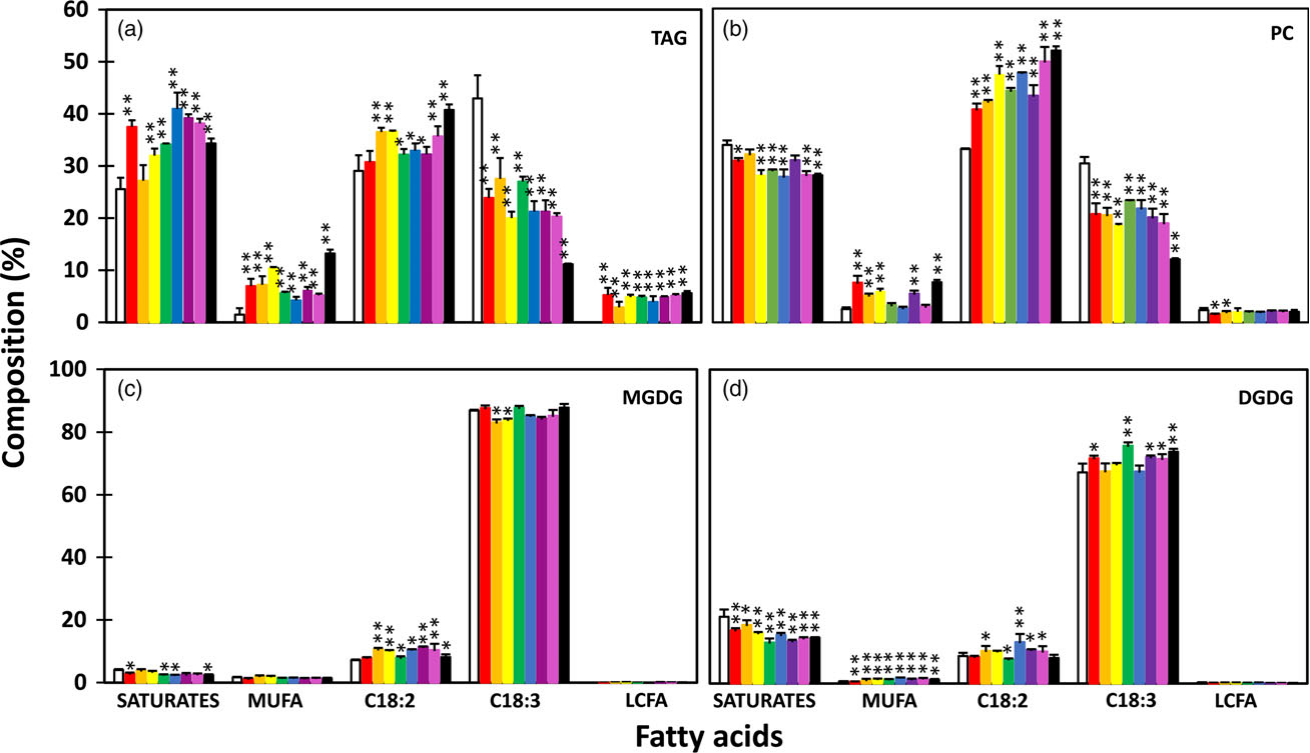
Fatty acid composition in triacylglycerol (TAG) (a), phosphotidylcholine (PC) (b) and monogalactosyldiacylglycerol (MGDG) (c) and digalactosyldiacylglycerol (DGDG) (d) in the leaves of wild type (WT) (white bar) and eight selected transgenic potato lines, including #17 (red bar), #47 (orange bar), #82 (yellow bar), #95 (green bar), #169 (blue bar), #69 (indigo bar), #149 (violet bar) and #68 (black bar). Asterisks indicate statistically significant differences between transgenic line and WT using Student's *t*‐test, with a significance threshold of 0.05 (*) and 0.01 (**). Error bars indicate standard deviations.

Significant increases in TAG content, ranging from 2‐ to 12‐fold, were also observed in fully expanded leaves at postanthesis stage in all the transgenic lines tested (Figure 7). The increases of PC, MGDG and DGDG were also evident, but to a much lesser extent compared to that of TAG. Interestingly, line #17 that showed the lowest TAG increase among all the eight transgenic lines showed the highest accumulation of GL (both MGDG and DGDG). Fatty acid composition of TAG in the transgenic leaves was also significantly altered (Figure 8a). ALA content was substantially reduced while the other fatty acids, including saturates, monounsaturates, LA and long‐chain fatty acids (LCFA, including C20:0, C22:0, C24:0 and C20:1), were increased. Compared to WT, saturates in PC and GL were significantly reduced, rather than increased as in TAG (Figure 8b–d). The substantial increase in LCFA was not observed in PC and GL.

**Figure 9:**
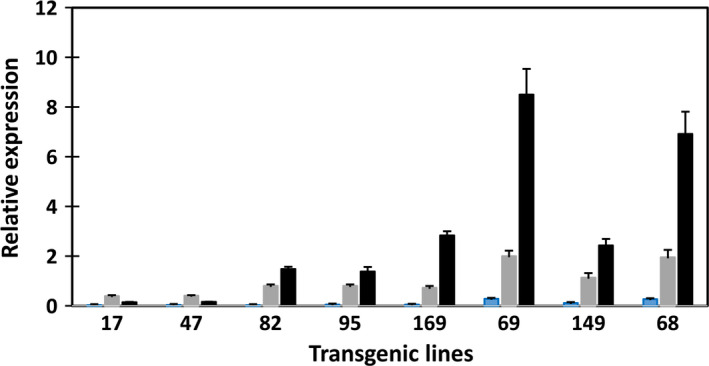
Real‐time qRT‐PCR analysis of transgene expression in transgenic potato leaves. *atWRI* (blue bar), *atDGAT1* (shade bar) and *siOLEOSIN* (black bar).

The expression levels of the three transgenes were also assessed with qRT‐PCR in transgenic potato leaves (Figure 9). Expression of *atDGAT1* driven by CaMV‐35S promoter was evident in leaves of the eight transgenic lines, with relatively high expression levels shown by lines #68, #149 and #69, coincidental with their relatively higher levels of TAG accumulation in leaf among the eight transgenic lines. Despite the expressions of *atWRI1* and *siOLEOSIN* being transcriptionally controlled by the patatin B33 promoter, *atWRI1* expression was also clearly detectable in the leaves of the above‐mentioned three high‐lipid lines, while various levels of *siOLEOSIN* expression were shown in all the transgenic leaves. Similar to the expression in tubers, *siOLEOSIN* expression level was the highest among the three transgenes in transgenic potato leaf.

### Analysis of sugar content, starch content and starch granule morphology of transgenic potato tuber

**Figure 10:**
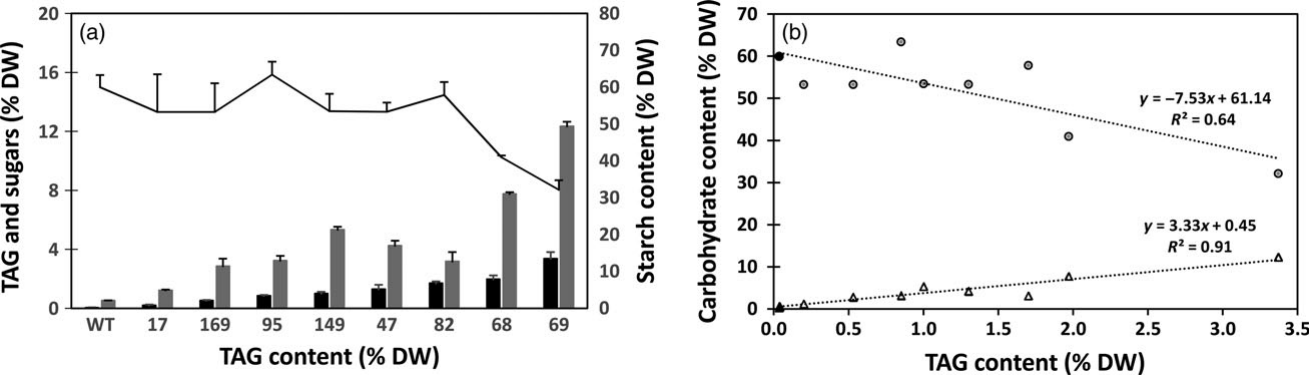
Carbohydrate accumulation in transgenic potato tubers. Contents of triacylglycerol (TAG) (black bar), soluble sugars (shade bar) and starch (line) in potato tubers (a). Relationship between TAG and carbohydrate content in wild type (WT) and transgenic potato lines (b): WT tuber (black symbols) has high starch (circles) and low soluble sugars (triangles) compared to transgenic potato lines (open symbols). Overall, the relationship between TAG and starch is negative (*y* = −7.53x + 61.14, *R* ^2^ = 0.64), while the relationship between TAG accumulation and soluble sugars is positive (*y* = 3.33x + 0.45, *R* ^2^ = 0.91).

**Figure 11:**
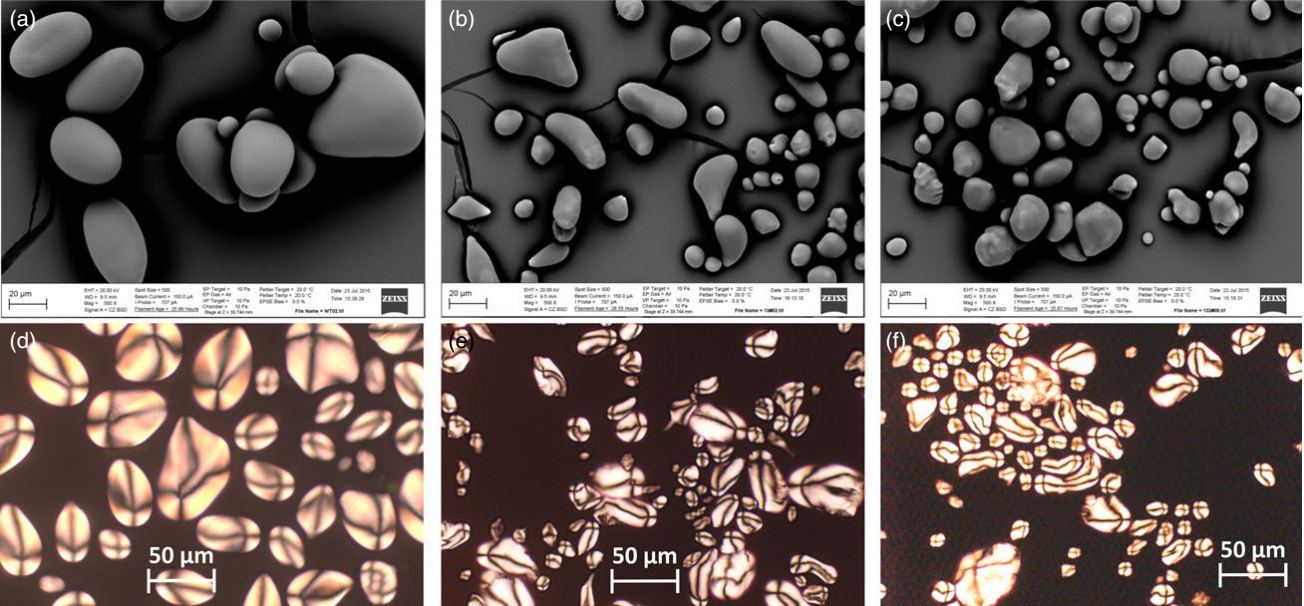
Starch granule morphology. SEM images of starch granules of wild type (WT) (a), transgenic lines #47 (b) and #69 (c). polarised light image of starch granules of WT (d), transgenic lines #47 (e) and #69 (f).

Significant increase in soluble sugar content and reduction in starch content were observed in transgenic lines compared to WT (Figure 10a). Such a trend in the change of carbohydrate accumulation was in a good correlation with the increase in TAG (Figure 10b). Scanning electron microscopy (SEM) revealed morphological differences of starch granules between WT and high‐lipid transgenic tubers, which are represented by lines # 47 and #69 (Figure 11). In contrast to the large, smooth‐surfaced ellipsoidal starch granules in WT (Figure 11a), the starch granules in the high‐lipid lines had angular and irregular shapes with some granules showing indentations on the surface (Figure 11b,c). However, birefringence (a measure of the level of crystalline order in starch granules) did not appear to be affected in the high‐lipid lines (Figure 11d–f) and all starch granules showed a distinct Maltese cross with similar brightness when examined by polarised light in both WT (Figure 11d) and transgenic lines (Figure 11e,f).

## Discussion

Potato tubers are developed from underground stems. Like most other underground storage organs, potato tubers accumulate mostly starch that is used as energy reserves for sprouting and early plant establishment. Potato tubers also accumulate storage proteins, predominantly the glycoprotein patatin (Hoefgen and Willmitzer, 1990), but have very low levels of TAG. This is in sharp contrast to an oilseed where TAG is the major energy reserve supporting seed germination until the seedling is capable of photosynthesis.

In this study, we have demonstrated that TAG levels, and to a lesser extent PL and GL levels, can be raised in potato tubers by coordinated up‐regulation of fatty acid production, TAG assembly and TAG packaging. This is an extension of the so‐called Push, Pull and Protection strategy into an underground tuber crop, a concept developed in transgenic tobacco leaves where a massive increase in TAG content (up to 15% of DW) was observed by co‐overexpressing *WRI1*,* DGAT1* and *OLEOSIN* genes (Vanhercke *et al*., 2014).

Yellow nutsedge (*Cyperus esculentus* L.) is perhaps the only plant known to accumulate high levels of dry matter (58%) and TAG (20%–36% DW) in tuber, which is comparable to a typical seed (Linssen *et al*., 1989; Turesson *et al*., 2010). But the utilisation of nutsedge tuber for vegetable oil production is not yet realistic because of its lack of domestication and environmental concerns. It is estimated that the transgenic potato with 25% dry matter and 3.3% TAG on DW basis could yield 393 kg vegetable oil per hectare in the USA where 47.7 tonnes potato fresh weight per hectare could be produced (FAOSTAT, http://faostat3.fao.org), which is comparable to current cotton seed oil yield.

The present day knowledge of fatty acid biosynthesis and TAG accumulation in plants is mainly derived from studies on seeds in Arabidopsis, and other oilseed crop species, while much remains unknown in nonseed plant tissue, such as potato tuber. In model plants, it has been established that WRI1 is a positive regulator of genes encoding enzymes involved in late glycolysis and fatty acid biosynthesis (Cernac and Benning, 2004; Chapman and Ohlrogge, 2012). It has two APETALA2 domains by which it binds to the promoter of the target genes, thereby activating and enhancing their transcription levels. *In vitro* experiments have demonstrated that WRI1 binds directly to the AW box of several fatty acid synthesis genes, and the mutation of the AW box abolished the WRI1‐mediated transcriptional activation in transient protoplast assays (Maeo *et al*., 2009). Expression studies have shown that target genes of WRI1 may include those encoding the BCCP subunit of ACCase, ACP, enoyl‐ACP reductase, β‐ketoacyl‐ACP reductase, plastidial pyruvate kinase, pyruvate dehydrogenase and FAD2 (Baud *et al*., 2007; Ruuska *et al*., 2002; To *et al*., 2012).

In potato, over‐expression of *ACCase* from Arabidopsis led to an increase in fatty acid biosynthesis and over fivefold increase in TAG content in tubers (Klaus *et al*., 2004). The identification of BCCP subunit of ACCase as a target gene of *WRI1* up‐regulation could be one of the key underlying mechanisms for TAG biosynthesis, as the result of *WRI1* over‐expression (Sasaki and Nagano, 2004). In maize, the expression of either *WRI1* or its upstream regulator *LEC1* was able to increase the oil content in seeds, but *WRI1* is advantageous as it does not give rise to detrimental effects such as poor seed germination, and stunted growth as seen in *LEC1* over‐expressing lines (Shen *et al*., 2010). In addition to developing seed, a *WRI1* homologue from oil palm has been shown to be highly expressed in oil‐accumulating mesocarp, 57‐fold higher than the closely related date palm that does not accumulate oil (Bourgis *et al*., 2011). Likely, as a result of the *WRI1* up‐regulation, the expressions of genes involved in *de novo* fatty acid biosynthesis have been found elevated in oil palm by 13‐fold, compared to date palm, while the transcripts of the genes involved in TAG assembly were similar between oil and date palm mesocarps (Bourgis *et al*., 2011). Subsequent experiments demonstrated that the oil palm *WRI1* homologue was able to rescue the Arabidopsis *wri1‐1* (Ma *et al*., 2013). This is regardless of the fact that the target gene *ACCase* in monocot species is highly divergent from that of dicot species. In the present study with potato tuber and leaf, the expression of *atWRI1* in transgenic potato tuber appeared to be moderate compared to *atDGAT1* and *siOLEOSIN,* which indicates a possible selection against those with high *WRI1* expression, and therefore, we have never recovered those tubers with very high level of *WRI1* expression. It is also possible that being a transcription factor gene, *WRI1* expression is normally at low levels compared to those encoding catalytic enzymes.

The expression of *DGAT1 and OLEOSIN* is unaffected by *WRI1* over‐expression in either Arabidopsis or maize (Maeo *et al*., 2009). Further, the peak expression of *DGAT* and *OLEOSIN* occurs much later compared to that of the *WRI1* during seed development (Kilaru *et al*., 2015). It is therefore necessary to co‐express *WRI1* together with *DGAT1* and *OLEOSIN* for an efficient enhancement of TAG production in transgenic plants, as we have demonstrated in tobacco leaf (Vanhercke *et al*., 2014) and the potato tuber reported herein.

DGAT1 catalyses the final step in the Kennedy pathway for TAG biosynthesis, which is considered as a rate limiting step in oilseeds (Jako *et al*., 2001; Lung and Weselake, 2006). The line #69 with the highest TAG accumulation also showed the highest *atDGAT1* expression in transgenic tubers; hence, the expression level of *atDGAT1* is a clear contributor to the high level TAG accumulation in potato. However, it is not clear how much of the TAG increase in potato tubers is attributable to the over‐expression of *DGAT1* which is under the transcriptional control of the CaMV‐35S promoter.

Both *atWRI1* and *siOLEOSIN* driven by patatin promoter have been found to be expressed in potato leaves, contrary to our initial anticipation of their tuber‐specific expression. The enhancement of lipid accumulation in potato leaves is therefore unlike a sole effect of CaMV‐35S driven *atDGAT1*. Perhaps at least partly due to some low level transcriptional activity of patatin promoter in leaf, the observed 12‐fold increase in TAG is far less than what was previously observed in tobacco leaves (Vanhercke *et al*., 2014). It has been previously reported that the expression of patatin is modulated by exogenous sucrose and DNA regions termed as sucrose responsive elements (SURE) elements have been identified in the proximal regions of the patatin promoter (Grierson *et al*., 1994). The unexpected raise of soluble sugars in the high‐lipid potato may have contributed to the leakiness of patatin promoter and led to TAG increase in potato leaf tissues.

DGAT1 uses DAG as a substrate that is also used by choline phosphotransferase to produce PC. In both potato tuber and leaf tissues, we have observed the concomitant increases of TAG and PC, rather than the increase in TAG at the expense of PC as shown in tobacco leaves (Vanhercke *et al*., 2014). Clearly, the increase in PC is necessary in the potato cells with dramatically increased TAG to maintain its sufficient presence on the LD surface. However, it is not yet known what physiological impact the substantial increase in polar lipids, especially GL, might have in transgenic potato tubers.

Recently, plant oleosin and other lipid body‐associated proteins have emerged as a new target for metabolic engineering of enhanced production in oil seeds as well as vegetative plant tissues. At least 17 differentially expressed *OLEOSIN* genes have been identified in Arabidopsis, suggesting that these genes are highly regulated even within a single plant species (Hsieh and Huang, 2004). The sesame *OLEOSIN* that was used in this study was previously demonstrated to enhance TAG accumulation in tobacco leaves (Vanhercke *et al*., 2014; Winichayakul *et al*., 2013). This is despite the fact that in Arabidopsis and maize the down‐regulation of the *OLEOSIN* expression did not lead to a decrease in oil accumulation (Siloto *et al*., 2006; Ting *et al*., 1996). In this study, a high level expression of the introduced *OLEOSIN* gene was associated with the highest TAG accumulation in transgenic potato.

A previous study revealed that the *OLEOSIN*s are not highly expressed in nonseed oleaginous tissues such as the mesocarp of olive, oil palm and avocado (Kilaru *et al*., 2015). Instead, lipid droplet‐associated proteins (LDAP) have been identified in these tissues that were associated with LD formation (Horn *et al*., 2013). It is therefore likely that oleosin may not be the optimal packaging protein in nonseed tissues to protect the accumulated oil from TAG lipase or other cytosolic enzyme activities. Additional research evaluating plant LDAP will be of particular interest in the further enhancement of TAG accumulation in potato tubers.

The observation of an increase in the number and size of LDs in transgenic potato indicated that the formation of LDs is clearly driven by the availability of TAG. A tight association of LDs with the ER has been observed in transgenic potato tubers, consistent with the hypothesis that LDs are formed on the surface of ER membrane (Thiam *et al*., 2013). It can be envisaged that an expanded ER network dedicated to the lipid production was formed in the transgenic potato tuber cells to cope with the increased TAG production. Based on studies in yeast and mammalian cells, it has been proposed that large LDs can arise either by LD expansion or fusion (Martin and Parton, 2006). With the supply of excess fatty acids, LDs in yeast cells may grow up to 30‐fold in volume within hours (Krahmer *et al*., 2011). In this study, LD fusion was not observed despite examination of a large number of TEM specimens. We may therefore assume that during LD growth, TAGs are added to the droplet cores and PLs to the surfaces. The close association between the ER and LDs may facilitate the transfer of the newly synthesised TAG to the droplet core.

To determine whether the enhanced lipid accumulation has impacted on the other major carbon reserve in potato, we quantified the starch and soluble sugar levels in the tubers of a number of selected transgenic potato lines. It appears that the introduction of an engineered lipid biosynthesis pathway overexpressing *WRI1/DGAT1/OLEOSIN* triple gene cassettes may have negatively affected starch accumulation in most of the high‐lipid lines. This is consistent with our previous observation with tobacco leaves where the up‐regulation of fatty acid biosynthesis and TAG assembly pathways has resulted in substantial reduction of starch accumulation (Vanhercke *et al*., 2014). In maize, the over‐expression of a *WRI1* gene resulted in increased oil content in the grains by 10%–22%, with a concomitant 60% reduction in starch content (Shen *et al*., 2010).

In our transgenic potato lines, consistent correlations were observed between TAG accumulation, starch reduction and soluble sugars accumulation, despite a few high‐lipid lines where the starch content was not significantly reduced. Enhancement of the lipid biosynthesis pathway in transgenic potato tubers likely had a detrimental effect on starch synthesis, leading to carbon accumulation in the form of soluble sugars. However, the relationship between starch and lipid accumulation in potato does not appear to be a simple redistribution of carbon but rather a complex mechanism that depends on the strategy used to reroute the carbon. For example, antisense down‐regulation of the starch biosynthetic enzyme AGPase in potato tubers resulted in a reduction of starch and an increase in soluble sugars but not fatty acids or TAG levels (Klaus *et al*., 2004). Although the underlying mechanism behind the decreased starch content in our study was not determined, it is likely the result of competition for carbon from the boosted lipid metabolic pathway, lowering the amount of metabolites available to be utilised for starch biosynthesis.

The hypothesis that competition for carbon decreases starch biosynthesis is consistent with the altered size and shape of starch granules in the transgenic potato tubers. Similar reductions in starch granule size, irregular granule shape and uneven surface morphology have been observed in several studies where the starch synthesis pathway was altered (Jobling *et al*., 2002; Regina *et al*., 2010; Shaik *et al*., 2016). However, in our case, preliminary investigation did not suggest any apparent change in starch birefringence or crystallinity. Further research is underway to investigate whether altered amylopectin/amylose composition is responsible for the observed changes in the size and morphology of starch granules in the transgenic potato tuber lines.

The fatty acid composition of polar lipids in potato tubers may reflect the biophysical features of the membrane structures of the cells and subcellular organelles. The degree of unsaturation of fatty acids in the constituent lipids of membranes is a major factor responsible for cell membrane fluidity at a given ambient temperature. We have observed a significant alteration of fatty acid composition in transgenic tubers, showing higher levels of oleic acid and LA, but lower levels of palmitic acid and ALA when compared to WT tubers. Our previous work with transgenic tobacco leaf showed a similar trend of alteration in fatty acid composition, but with more dramatic increase in oleic acid (Vanhercke *et al*., 2014). This could be as the result of WRI1 regulation on the expression of microsomal fatty acid desaturases as previously suggested by Ruuska *et al*. (2002). In transgenic tobacco leaf, the expression levels of *FAD3* and *FAD7* responsible for the ALA production in eukaryotic and prokaryotic pathways were found to be down‐regulated, leading to the reduction of trienoic fatty acids (Vanhercke *et al*., 2014). WRI1 was also shown to transactivate the *FATTY ACID ELONGASE1* gene (*FAE1*), and an AW box was also located in the promoter region of *FAE1* (Maeo *et al*., 2009). This might partly explain our observation of a substantial increase in LCFAs in both tuber and leaf of the transgenic potato lines with increased TAG accumulation in this study. This is consistent with the characterisation of seed oil in Arabidopsis *wri1* mutant that featured higher proportions of ALA and to a lesser extent, erucic acid (C22:1^Δ13^) at the expense of oleic acid, LA and eicosenoic acid (C20:1^Δ11^) (Cernac and Benning, 2004). It might also be possible that an enhanced DGAT activity may have selectively increased the flux of linoleoyl‐DAG to TAG and deprived it of opportunity for further desaturation while associated with PC. In maize, a high‐oil QTL with increased TAG content as the result of a single amino acid substitution in DGAT1 also showed significantly raised oleic acid contents at the expense of polyunsaturates (Zheng *et al*., 2008). The fatty acid profile of lipids has important implications for the functioning of biological membranes as well as for postharvest applications. Further research should be conducted on the plant physiology and adaptations to different growth temperatures by the transgenic potato with altered lipid profile. It could be envisaged that such an alteration in fatty acid composition is favourable for food applications, as the high ALA content in WT tubers leads to higher levels of autoxidation that is largely responsible for off‐flavours and rancidity problems in processed potato food products. Galliard (1970, 1972) demonstrated that the presence of phospholipase, galactolipase and lipoxygenases could lead to rapid lipid peroxidation when tuber cells were broken. Together with the increase in saturated fatty acids, reduction in the level of ALA is anticipated to have marked effects on the improvement of oxidative stability of potato products. Further, the change of fatty acid composition is also favourable for biodiesel applications due to the improved oxidative stability, and possibly ignition quality and cold‐temperature flow properties in potential biofuel applications (Rogalski and Carrer, 2011).

## Concluding remarks

In this study, we have demonstrated as a proof of concept that potato tubers could be used as a potential target storage organ for TAG accumulation through a synergistic engineering approach, providing a basis for further optimisation of the ‘Push’, ‘Pull’ and ‘Protect’ strategy to further enhance TAG accumulation in a nonphotosynthetic underground sink organ. The moderately enhanced oil accumulation in potato tubers demonstrated herein should encourage further studies of TAG biosynthesis and turnover, and the intricate relationship between oil and starch in a classic starch‐accumulating storage organ. Because the TAG content reaches more than 30% of DW in an anatomically similar nutsedge tuber, at least in theory it might be possible to further raise the TAG level in transgenic potato tubers. Increased knowledge of the lipid accumulation mechanism in transgenic potato would generate opportunities to adapt the large biomass tuber potato crop to meet the global food challenges of lipid production. There is also an opportunity to introduce novel fatty acids with health benefits or high industrial values to high‐lipid tubers. Such ‘dual purpose’ crop strategies may have potential for direct use as a niche health food, animal feed or oleochemical feedstock.

## Experimental procedures

### Binary plasmid construct pJP3506

A binary plasmid construct, pJP3506, was derived from a pORE04‐based binary expression vector, which contained *NPTII* gene driven by double enhancer CaMV 35S promoter (e35S) as the selectable marker and three gene expression cassettes, which are *35S::atDGAT1*,* B33::atWRI1* and *B33::siOLEOSIN*, respectively. Patatin B33 promoter derived from *S. tuberosum* was kindly provided by Dr Alisdair Fernie, Max Planck Institute of Molecular Plant Physiology in Germany. It is a truncated version with 185 bp deleted from the 5′ end of the published GenBank sequence (GenBank accession number X14483). *AtWRI1* is the codon‐optimised coding region of *A. thaliana WRI1* gene, and *atDGAT1* referred to the WT *A. thaliana DGAT1* gene and *siOLEOSIN* is an intron‐interrupted *S. indicum OLEOSIN* gene as previously described in Vanhercke *et al*. (2014). A diagram showing the configuration of the arrangement of the transgene cassettes in pJP3506 is presented in Figure 1.

### Potato transformation

*In vitro* seedlings of commercial potato cultivar, *S. tuberosum* cv. Atlantic were purchased from Toolangi Elite^™^, Healesville, Victoria, Australia. Stem internodes were excised into approximately 1‐cm pieces in length under *Agrobacterium tumefaciens* strain AGL1 (OD_600_ = 0.2) grown in a MS and LB mixture media (50 : 50, by volume). Following a brief blotting on sterile Whatman filter paper, the infected internodes were plated out onto MS media agar plates supplemented with 200 μg/L NAA and 2 mg/L BAP, and maintained at 24 °C. Following 2 days co‐cultivation and further 8 days on fresh medium without selection, the explants were transferred onto MS medium supplemented with 2 mg/L BAP, 5 mg/L GA_3_, 50 mg/L kanamycin (for transgenic selection) and 250 mg/L cefotaxime (to control Agrobacterium) until the emergence of green shoots which were excised and placed onto plain MS medium for root induction prior to transplanting into a 20‐cm diameter pot containing potato potting mix (CSIRO). The plants were maintained at a greenhouse with 25/20 °C with extended light for 16‐h photoperiod. The tubers harvested from the primary transgenic plants were used for initial screening, and selected eight lines, together with untransformed potato, were grown at the same condition for further analysis.

### RNA extraction and transgene expression profiling in transgenic plants by qRT‐PCR

Total RNAs from developing potato tubers at about 4 cm in diameter and fully expanded green leaves at the same stage were isolated using RNeasy Mini Kit (Qiagen, Hilden, Germany). Contaminating DNA was removed by digestion with Ambion TURBO RNA‐free DNaseI (Thermo Fisher Scientific, Waltham, MA) according to the manufacturer's protocol. RNA concentrations were determined using a Nanodrop^®^ spectrophotometer ND1000 (Thermo Fisher Scientific), and concentrations were standardised before analysis. To verify the RNA integrity, 1 μg of total RNA from each sample was visualised on an ethidium bromide‐stained 1.5% agarose gel following electrophoresis.

The gene expression patterns were studied with qRT‐PCR in triplicate using Platinum SYBR Green qPCR SuperMix (Bio‐Rad, Hercules, CA) and run on ABI 7900HT Sequence Detection System. Each PCR contained 20 ng of total RNA template, 50 pmol each of the forward and reverse primers, 0.25 μL of reverse transcriptase, 5 μL One‐step RT‐PCR master mix reagents, increased to 10 μL total volumes with nuclease‐free water. The primers for *atWRI1* are sense: 5′‐CTCCAACTACATCGACAGGC ‐3′; antisense: 5′‐ GCAAGGTAGTAGAGGACGAAG‐3′. The primers for *atDGAT1* are sense: 5′‐GGCGATTTTGGATTCTGCTGGC‐3′; antisense: 5′‐GGAACCAGAGAGAAGAAGTC‐3′; and the primers for sesame *OLEOSIN* are sense: 5′‐CAGCAGCAACAAACACGTG‐3′; antisense: 5′‐GAGAAGATCACCAGGAGAG‐3′. A constitutively expressed *S. tuberosum* gene *CYCLOPHILIN (stCYP)* was used as the reference gene to normalise the relative quantities (GenBank accession number AF126551; Nicot *et al*., 2005). The oligo sequence of the primers for *stCYP* is sense: 5′‐CTCTTCGCCGATACCACTC‐3′; and antisense: 5′‐CACACGGTGGAAGGTTGAG‐3′. The thermal cycling conditions were reverse transcription at 45 °C for 10 min, following deactivation by 95 °C for 2 min. This was followed by 40 cycles of 95 °C for 5 s and 60 °C for 50 s. The calculations were made using the comparative CT method (2^−ΔΔCt^) as reported (Livak and Schmittgen, 2001).

### Lipid analysis

Fresh tubers were harvested from potato plants at full maturity when the up‐ground stems and leaves turned brown and dead. A thin slice was sampled from the middle of the tuber and freeze‐dried for 72 h prior to lipid analysis. Full sized green potato leaf tissues were sampled for lipid analysis when the potato plant approach maturity and two‐thirds of the leaves had turned brown. To extract the total lipids, the freeze‐dried tuber tissues were first homogenised in chloroform/methanol (2 : 1, by volume) in an eppendorf tube containing a metallic ball using the TissueLyser (Qiagen) and mixed with one‐third volume of 0.1 m KCl. Following centrifugation at 10 000 ***g*** for 5 min, the lower liquid phase containing lipids were collected and evaporated completely using N_2_ flow before 3 μL of chloroform was added for each milligram of tuber DW. Lipid samples prepared as above were loaded on a TLC plate (10 cm × 20 cm, Silica gel 60; Merck, Darmstadt, Germany) and developed in hexane : diethyl ether : acetic acid (70 : 30 : 1, by volume). The TLC plate was sprayed with primuline made in 80% acetone in water and visualised under UV. TAG and PLs were fractionated by cutting out the corresponding silica bands, and their respective fatty acid methyl esters (FAME) were produced by incubating corresponding silica bands in 1 N methanolic HCl (Supelco, Bellefonte, PA, USA) at 80 °C for 2 h together with triheptadecanoin (Nu‐Chek PREP, Inc., Elysian, MN) as internal standard for lipid quantification. FAME were analysed by gas chromatography (7890A GC; Agilent Technologies, Santa Clara, CA) equipped with a 30 m BPX70 column (0.25 mm inner diameter, 0.25 mm film thickness; SGE, Austin, TX). Peaks were integrated with Agilent Technologies ChemStation software (Rev B.04.03).

### Quantification of starch and soluble sugars

Fresh mature potato tubers were sampled for carbohydrate analysis when the up‐ground stems and leaves had turned brown and dead. Triplicate samples (~10 mg) of ground freeze‐dried potato tuber tissue were boiled four times in 80% ethanol. The supernatants (ethanol extract) were pooled for analysis of soluble sugars, while the pellet was retained for starch determination.

Aliquots of ethanol extract were boiled in anthrone reagent (2% anthrone in 70% H_2_SO_4_, by volume) for 10 min, and the absorbance at 630 nm was measured for the analysis of soluble sugar contents (Yemm and Willis, 1954). For starch determination, the pellet was resuspended in 350 μL 0.2 m NaOH, boiled for 30 min and neutralised using 3.5 μL glacial acetic acid. The starch content was determined relative to a control/blank aliquot using a Megazyme Total Starch Kit following the manufacturers’ instruction (Megazyme International Ireland, Bray, Ireland). All spectrophotometric measurements were performed using a Thermo Multiskan^®^ Spectrum plate reader (Thermo Fisher Scientific).

### Observation of LDs and confocal fluorescence microscopy imaging

Thin slices of the freshly harvested immature potato tubers at about 4 cm in diameter were fixed in 4% paraformaldehyde, prior to embedding in CRYO‐GEL^™^ embedding medium (Instrumedics Inc., Hackensack, NJ). The mounted samples were then frozen at −20 °C for 48 h prior to sectioning with a microtome. Glass microscope slides containing potato sections of 40 μm in thickness were stained with 1 μg/mL solution of Nile Red (Sigma‐Aldrich, St. Louis, MO) and imaged using a Leica SP8 confocal scanning microscope (Leica Micrisystems, Wetzlar, Germany). The excitation wavelength was 488 nm, and the emission was recorded between 593 and 654 nm. LDs were imaged at 40× magnification and analysed using Leica LAS AF Lite software (www.leica-microsystems.com).

### Isolation of starch and light and scanning electron microscopy imaging

Starch granules were isolated from mature potato tuber using a method adapted from Wischmann *et al*. (2007). Peeled potatoes were homogenised in 1% (w/v) sodium metabisulphite, and the resulting mixture was filtered through 200 μm nylon mesh. The slurry was steeped for at least 40 min to allow the starch to settle. The pelleted starch was repeatedly washed with and steeped in deionised water until the wash was clear. Purified starch was then washed three times with deionised water and dried overnight prior to observation of starch granules with light microscopy (Leica‐DMR) using crossed polarised filters to reveal birefringence in the starch granules at a 400× magnification. Starch granule morphology was also examined with a scanning electron microscope (ZEISS EVO LS15, Carl Zeiss International, Oberkochen, Germany). Purified starches were sputter‐coated with gold and scanned at 20 kV at room temperature at 500× magnification.

### Transmission electron microscopy imaging

Freshly harvested immature potato tubers at about 4 cm in diameter were cut into approximately 2 × 1 mm pieces and directly fixed with 2.5% glutaraldehyde, 2% paraformaldehyde in 0.1 m phosphate buffer (pH 7.4), under vacuum for 24 h. The samples were then washed with 0.1 m phosphate buffer (pH 7.4) three times and for 10 min each. The secondary fixation with 1% osmium tetroxide was carried out at room temperature for 4 h. The fixed samples were rinsed with distilled water and dehydrated through an acetone series and embedded in Spurr's resin (Spurr, 1969) overnight at 70 °C. Subsequently, ultrathin sections (70–90 nm) were obtained with a Leica EM UC7 Ultra microtome. Sections were stained with uranyl acetate and lead citrate for 10 min each. Images were observed and recorded using the Hitachi 7100 TEM (Hitachi High Technologies America, Inc., Schaumburg, IL) at an accelerating voltage of 100 kv.

### Statistical analysis

Significance of differences in the biochemical measurements of soluble sugars, starch and lipids was determined using ANOVA and student *t*‐test. Differences were considered as statistically significant when *P* < 0.05 (represented by ‘*’) and highly significant when *P* < 0.01 (represented by ‘**’), compared with the control group.
